# Indoor Application of Attractive Toxic Sugar Bait (ATSB) in Combination with Mosquito Nets for Control of Pyrethroid-Resistant Mosquitoes

**DOI:** 10.1371/journal.pone.0084168

**Published:** 2013-12-19

**Authors:** Zachary P. Stewart, Richard M. Oxborough, Patrick K. Tungu, Matthew J. Kirby, Mark W. Rowland, Seth R. Irish

**Affiliations:** 1 Faculty of Infectious Tropical Diseases, London School of Hygiene and Tropical Medicine, London, United Kingdom; 2 Department of Agronomy and Horticulture, University of Nebraska, Lincoln, Nebraska, United States of America; 3 Pan-African Malaria Vector Research Consortium (PAMVERC), Moshi, Tanzania; 4 Department of Parasitology and Entomology, Kilimanjaro Christian Medical University College (KCMUCo) of Tumaini University, Moshi, Tanzania; 5 Amani Medical Research Centre, National Institute for Medical Research, Muheza, Tanzania; 6 Malaria Consortium, London, United Kingdom; University of Crete, Greece

## Abstract

**Background:**

Attractive toxic sugar bait (ATSB) sprayed onto vegetation has been successful in controlling *Anopheles* mosquitoes outdoors. Indoor application of ATSB has yet to be explored. The purpose of this study was to determine whether ATSB stations positioned indoors have the potential to kill host-seeking mosquitoes and constitute a new approach to control of mosquito-borne diseases.

**Methods:**

Insecticides were mixed with dyed sugar solution and tested as toxic baits against *Anopheles arabiensis*, *An. Gambiae s*.s. and *Culex quinquefasciatus* in feeding bioassay tests to identify suitable attractant-insecticide combinations. The most promising ATSB candidates were then trialed in experimental huts in Moshi, Tanzania. ATSB stations were hung in huts next to untreated mosquito nets occupied by human volunteers. The proportions of mosquitoes killed in huts with ATSB treatments relative to huts with non-insecticide control treatments huts were recorded, noting evidence of dye in mosquito abdomens.

**Results:**

In feeding bioassays, chlorfenapyr 0.5% v/v, boric acid 2% w/v, and tolfenpyrad 1% v/v, mixed in a guava juice-based bait, each killed more than 90% of pyrethroid-susceptible *An. Gambiae* s.s. and pyrethroid-resistant *An. arabiensis* and *Cx. quinquefasciatus*. In the hut trial, mortality rates of the three ATSB treatments ranged from 41-48% against *An. arabiensis* and 36-43% against *Cx. quinquefasciatus* and all were significantly greater than the control mortalities: 18% for *An. arabiensis*, 7% for *Cx. quinquefasciatus* (p<0.05). Mortality rates with ATSB were comparable to those with long lasting insecticidal nets previously tested against the same species in this area.

**Conclusions:**

Indoor ATSB shows promise as a supplement to mosquito nets for controlling mosquitoes. Indoor ATSB constitute a novel application method for insecticide classes that act as stomach poisons and have not hitherto been exploited for mosquito control. Combined with LLIN, indoor use of ATSB has the potential to serve as a strategy for managing insecticide resistance.

## Introduction

Increased coverage of insecticide-treated nets (ITN) and indoor residual spraying (IRS) has been successful in reducing the malaria burden of many African countries. Despite this public health success there is concern with the over-dependency on the pyrethroid class of insecticides, and the implications of wide-scale selection of pyrethroid resistance on the future control of malaria [1]. By 2012, pyrethroid resistance in *Anopheles gambiae* s.l. [2,3] and *Culex quinquefasciatus* [4,5] had been reported across much of Africa. The negative impact of pyrethroid resistance on ITN effectiveness has been demonstrated in experimental hut trials [6] and household trials [7], although the point at which pyrethroid resistance translates to diminished control of malaria transmission has yet to be demonstrated [8,9]. In light of this, novel and complementary techniques are urgently needed to manage resistance, prevent malaria resurgence and to maintain the drive towards malaria elimination.

In practice most new insecticides in the last 30 years have been developed for the agricultural sector for their activity against phytophagous pests and for their non-persistence in the environment, whereas the best adulticides for mosquito control have been highly residual and act through cuticular contact. Consequently, there are modern classes of insecticide that are effective by ingestion, and show no sign of cross resistance, but which continue to be underutilized in public health [10]. If a suitable delivery system could be developed for such insecticides against adult mosquitoes, this could provide an important new method for disease transmission control, supplementary to pyrethroid-treated nets, particularly against mixed populations of susceptible and resistant mosquitoes. 

Both male and female mosquitoes use plant sugars as an energy source. *Anopheles gambiae s.s.* females exhibit a discriminative preference for plants with high glucose and fructose contents, and successful feeding from such plants correlates with high survival and egg laying rates in this species [11]. Attractive toxic sugar baits (ATSB) can take advantage of this behaviour to control mosquitoes by using a combination of a concentrated sugar-based food source, an olfaction stimulant and an oral insecticide to lure and kill mosquitoes at a bait station. Until now the application of ATSB as a mosquito control tool has been limited - albeit highly successfully - to outdoor use [12,13]. Because exposure to primary African malaria vectors still occurs largely indoors [14,15], a bait station delivery system deployed in the home, that is attractive and toxic to mosquitoes and easy-to-maintain could have wide application as a means of controlling pyrethroid resistant mosquitoes which might otherwise survive exposure to long lasting insecticidal nets [6,7]. 

The study described here was designed to assess the added benefit of using ATSB stations alongside mosquito nets to control wild populations of *An. arabiensis* and *Cx. quinquefasciatus*. Attractants and insecticides were first tested in laboratory bioassays and then candidate ATSB were evaluated in experimental huts.

## Materials and Methods

### ATSB laboratory bioassays

#### Insecticides

Three oral insecticides were tested: boric acid 2% w/v (Boric Acid 99.5%, Nairobi Labcare Ltd, Nairobi, Kenya), tolfenpyrad 1% v/v (OMI-88 15SC, Nihon Nohyaku Co. Ltd., Tokyo, Japan), and chlorfenapyr 0.5% v/v (Phantom SC 21.45%, BASF, Ludwigshafen, Germany). These insecticides were selected because they have different modes of action (inorganic stomach poison, mitochondrial electron transport inhibitors and oxidative phosphorylation uncouplers, respectively) and potential to kill pyrethroid-resistant mosquitoes had been previously demonstrated (Irish, unpublished data) [12,16,17]. Preliminary experiments determined appropriate concentrations of each insecticide.

#### Sugar baits

The sugar bait solution (SBS) used in the bioassay tests consisted of similar ingredients to those used in previous ATSB studies [13,18], namely 35% v/v guava juice (Azam, Bakhresa Food Products Ltd, Dar es Salaam, Tanzania), 10% w/v brown sugar and 2% v/v red food dye (Dr. Oetker, Leeds, UK). The guava juice was ripened for 48hrs in a closed container at ambient temperature before adding it to the bait solution. Guava juice is known to be highly attractive to *An. gambiae* [19] and was also found to be similarly attractive to *An. arabiensis* and *Cx. quinquefasciatus* in observational tests (Stewart, unpublished data).

#### Mosquitoes

Three different mosquito strains were evaluated in bioassays:

1. *An. gambiae* *sensu* *stricto* Kisumu strain, pyrethroid susceptible, originally from Kenya;
2. *An. arabiensis*, pyrethroid-resistant, F_1_ generation of mosquitoes collected from Lower Moshi, Tanzania;
3. *Cx. quinquefasciatus* Masimbani strain, pyrethroid-resistant, originally collected in Muheza, Tanzania.

#### Study design

The following treatment arms were compared:

1. SBS without insecticide;
2. SBS plus tolfenpyrad 1% v/v;
3. SBS plus chlorfenapyr 0.5% v/v;
4. SBS plus boric acid 2% w/v

ATSB solution (25ml) containing food dye was soaked into cotton wool pads placed on plinths in the center of 30cm sided mosquito cages. Fifty female mosquitoes, 3-4 days old, previously provided with sugar solution *ad libitum*, were denied access to sugar for four hours and then were introduced into each cage and left overnight. The following morning mosquitoes were scored as alive or dead and fed or unfed. Mosquitoes that fed on the solution were identified by the presence of food dye visible through the cuticle of the abdomen. Live mosquitoes were transferred to paper cups, provided with a 10% w/v glucose cotton pad, and held at 26°C and 70-80% humidity. No more than 10 mosquitoes were held in each cup. After 24 hours, mosquitoes were scored for delayed mortality. Three replicates of 50 mosquitoes of each species were tested against each treatment.

### Experimental hut study

#### Study site

Four experimental huts in Pasua village, Kilimanjaro, Tanzania (3°22’46S and 30°20’47E) were used for the field trial of ATSB. The site was situated next to flooded rice fields, surrounded by flowering plants, hedges and fruit trees, and with domestic animals and houses present within 50m of the huts. These verandah trap huts were built to a standard East African design as described by WHO for indoor evaluation of vector control products [20]. The huts were constructed of adobe bricks, plastered with mud, with roofs of corrugated iron. White linoleum flooring facilitated the collection of dead mosquitoes in rooms and verandas. The central room was surrounded by four verandas, two of which were screened to capture mosquitoes exiting through the 5cm eave gap between walls and ceiling. Mosquitoes could also exit into two traps placed over windows on two sides of the room. In the eaves of the two open verandas, wooden baffles funneled host-seeking mosquitoes into the room and prevented mosquitoes from exiting in the opposite direction through the open verandas. 

Previous studies have shown the *An. arabiensis* population in this area to be pyrethroid-resistant, due to elevated mixed function oxidases and esterases rather than *kdr* mechanisms [21]. 

#### Bait Stations

**Figure 1:**
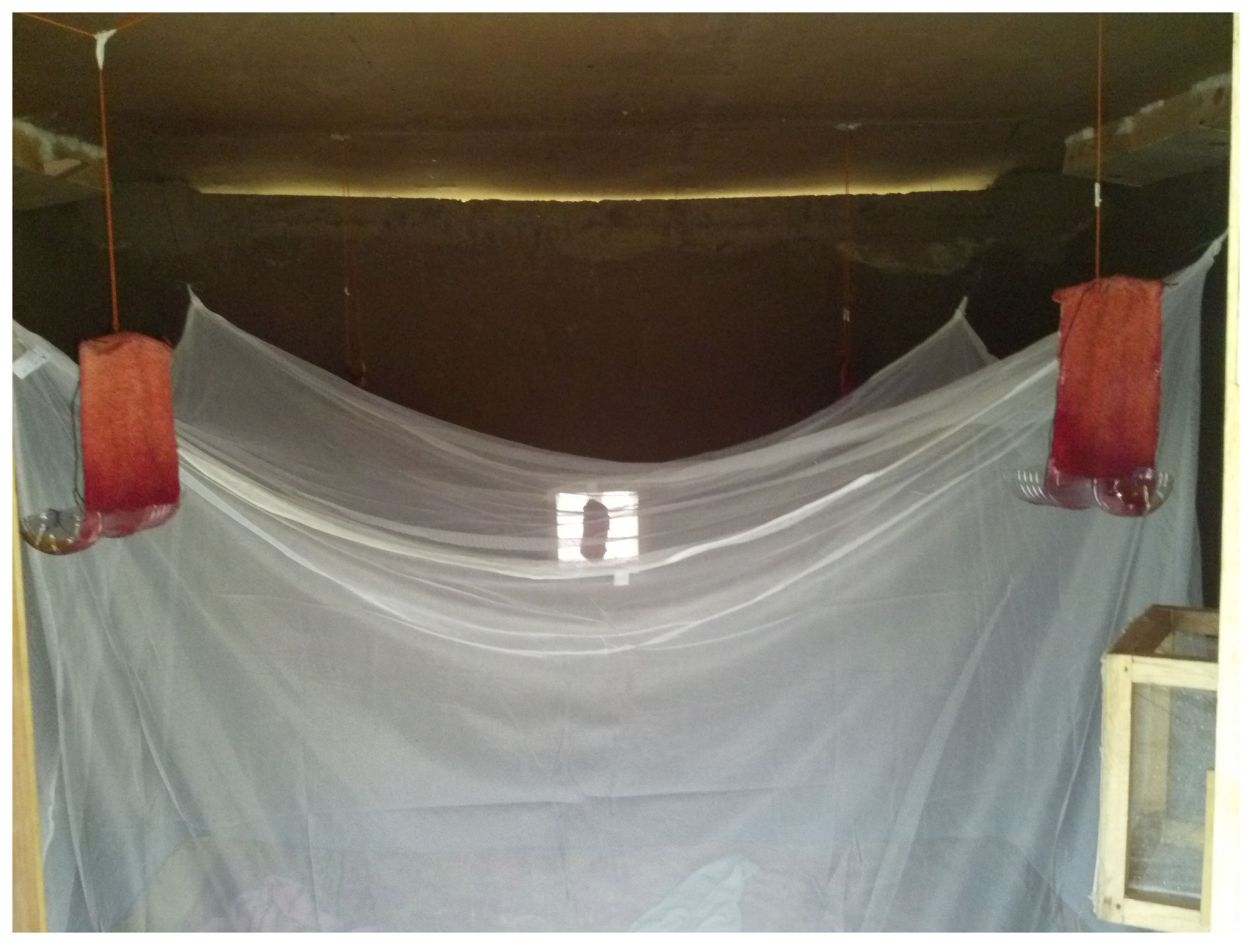
ATSB station positioning in experimental huts.

**Figure 2:**
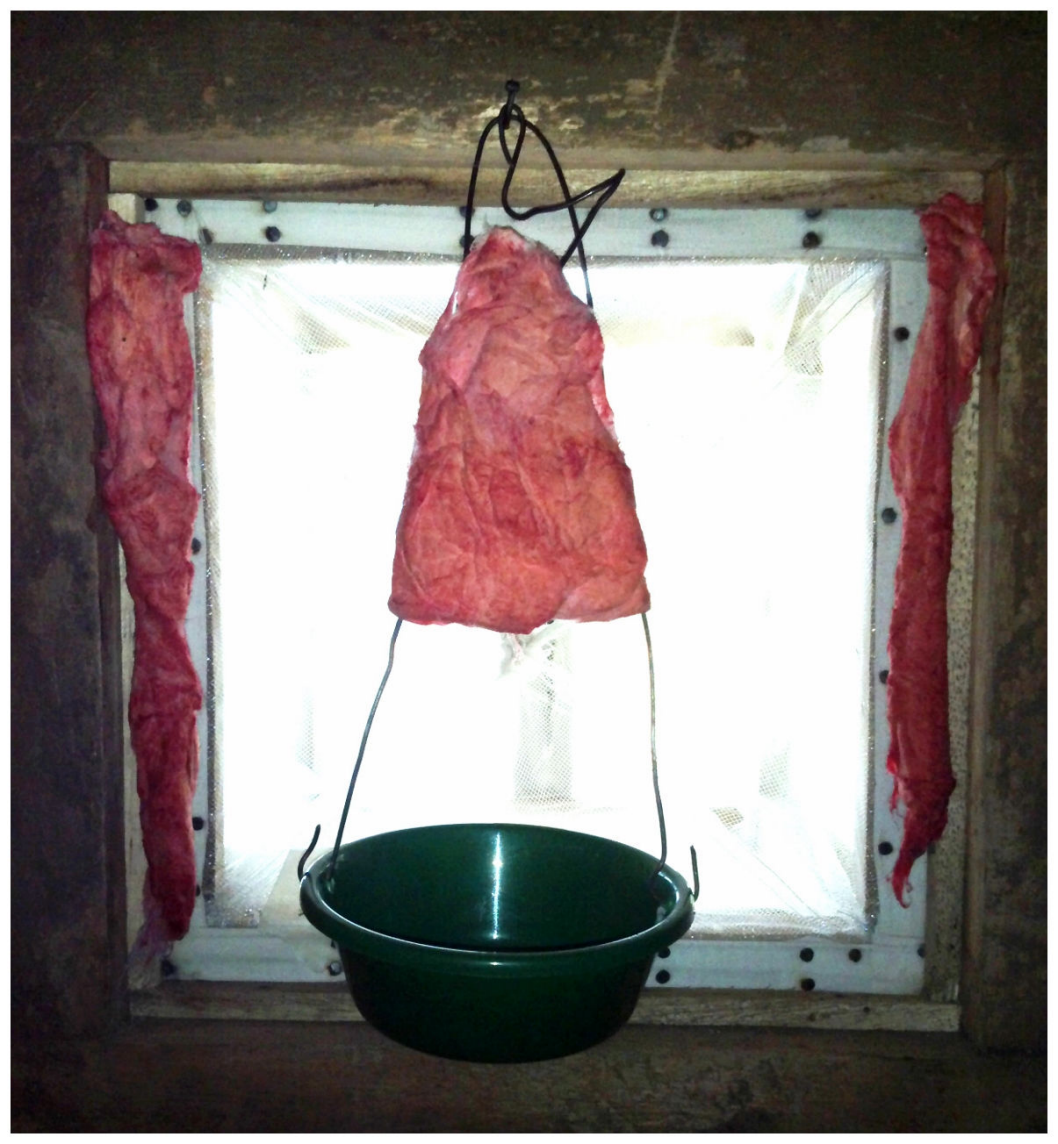
ATSB station positioning near window traps.

ATSB solution was soaked into cotton towels (20 x 30cm), attached to frames and positioned over plastic drip trays that served as reservoirs for re-absorption of solution by the towel wicks. Four of these bait stations were hung from the ceilings, approximately 150cm above the ground, at the corners of the untreated bed nets (Figure 1). Smaller cotton wool baits were positioned near each window trap, with one bait in the centre of the window and two bait strips on either side (Figure 2). ATSB solution was added each night to keep the bait stations moist. To reduce desiccation of mosquitoes which exited the huts without feeding on the ATSB, cotton wool pads soaked in 10% glucose (non-insecticidal) were placed inside the window traps and closed verandas of each hut to serve as water and energy sources. The control hut had the same arrangement of bait stations, but these were impregnated with sugar solution without insecticide.

#### Study Design

The trial ran for 16 nights between July 31 and August 19, 2012. An adult male volunteer slept in each hut under an unholed untreated mosquito net from 19:00 to 06:00h. The ATSB stations were rotated through the huts according to a Latin square design so that each treatment was trialed with each sleeper and hut for a contiguous period of 4 days. The same three treatments and control trialed in the laboratory bioassays were tested in the huts.

Each morning at 06:30h dead and live mosquitoes were collected from the veranda and window traps and dead mosquitoes were collected from the floors of the rooms for standardized periods by pairs of mosquito collectors who took it in turns to check each others’ territory. Live mosquitoes were deliberately left to exit from the rooms naturally. Mosquitoes were scored as live or dead, bait fed or not bait fed, and identified to species. Live mosquitoes were kept for delayed mortality assessment, adopting the same procedure as used in the choice tests. Bait-fed status (dye visible in abdomens) was double scored by two technicians.

### Data Analysis

Data was analyzed using Stata 12.1 software (Stata Co., College Station TX, USA). Analysis of the proportions killed and proportion feeding on the bait in laboratory bioassays was performed using logistic regression, adjusting for clustering by replicate test and whether or not dye was visible in the abdomens. Analysis of proportions killed in experimental huts and proportion feeding on the bait was performed using logistic regression, adjusting for the effect of sleeper and hut position. 

### Ethical Clearance

Approval was obtained from the London School of Hygiene and Tropical Medicine Ethics Review Committee (no. 011/391) and by the Tanzanian National Institute of Medical Research (NIMR/HQ/R.8c/Vol.I/24). Trial participants gave written informed consent to sleep under untreated nets, and were offered free medical services during and up to three weeks after the end of the trial.

## Results

### ATSB laboratory bioassays

All ATSB insecticide treatments resulted in high mortality rates of female *An. gambiae s.s.*, *An. arabiensis* and *Cx. quinquefasciatus* (Table 1). Chlorfenapyr 0.5%, boric acid 2% and tolfenpyrad 1% ATSB killed 100%, 85% and 86% of female *An. gambiae s.s.*, within 24h of testing compared to 13% mortality in the control (p<0.001). Similar trends were observed for *An. arabiensis* and *Cx. quinquefasciatus*, with mortality ranging from 83% to 99% between insecticide treatments. 

**Table 1:** Laboratory bioassays of three toxic sugar baits against *An. gambiae* s.s., *An. arabiensis*, and *Cx. quinquefasciatus* in cage tests. Numbers of female mosquitoes tested, the percentage dying, the percentage dyed with a red dye, and the percentage of dyed mosquitoes that died (and 95% confidence intervals) are presented.

			**Control**	**Chlorfenapyr (0.5%)**	**Boric acid (2%)**	**Tolfenpyrad (1%)**
***Anopheles gambiae*** females						
	**Total tested**		135	147	148	143
	**Mortality (%)**		13 (8-19)^a^	100^b^	85 (78-89)^c^	86 (79-91)^c^
	**Mosquitoes with visible dye (%)**		74 (66-81)^a^	28 (21-36)^b^	68 (60-75)^a^	26 (19-34)^b^
	**Mortality of visibly dyed mosquitoes (%)**		9 (5-16)^a^	100^b^	92 (85-96)^c^	100^b^
***Anopheles arabiensis*** females						
	**Total tested**		55	58	59	61
	**Mortality (%)**		4 (1-13)^a^	91 (81-96)^b^	83 (71-91)^b^	89 (78-94)^b^
	**Mosquitoes with visible dye (%)**		89 (78-95)^a^	45 (33-58)^b^	64 (52-76)^b^	41 (29-54)^b^
	**Mortality of visibly dyed mosquitoes (%)**		2 (0-6)^a^	100^b^	89 (78-94)^c^	100^b^
***Culex quinquefasciatus*** females						
	**Total tested**		157	153	165	166
	**Mortality (%)**		3 (1-7)^a^	95 (90-97)^b^	78 (71-83)^c^	99 (96-100)**^*d*^**
	**Mosquitoes with visible dye (%)**		85 (78-90)^a^	81 (74-87)^a^	86 (80-91)^a^	44 (37-52)^b^
	**Mortality of visibly dyed mosquitoes (%)**		2 (0-6)^a^	100^b^	89 (83-96)^c^	100^b^

If the superscript in a row is the same, there were no significant differences between the treatments (p>0.05)

For the control treatment the proportion of mosquitoes with dye visible in the abdomen was 74% for *An. gambiae*, 89% for *An. arabiensis* and 85% for *Cx. quinquefasciatus*. For the chlorfenapyr and tolfenpyrad treatments the proportions with visible dye was significantly lower (p<0.001) compared to the control across all three species (*Cx. quinquefasciatus* exposed to chlorfenapyr was the only exception). Comparing treatments, the proportion of mosquitoes with dye visible was always higher in the boric acid treatment than in chlorfenapyr and tolfenpyrad treatments (Table 1). Chlorfenapyr and tolfenpyrad ATSB always proved fatal to mosquitoes which had visible dye, whereas for boric acid 8-11% survived. 

### Experimental Hut Trial

Over the course of the 16 trap nights, 1374 mosquitoes were collected. Of these, 1170 were *An. arabiensis* and 204 were *Cx. quinquefasciatus*. Only 35% of the *An. arabiensis* collected were female, while 73% of the *Cx. quinquefasciatus* were female. The number of *An. arabiensis* females collected per treatment ranged from 97 to 109, the number of *Cx. quinquefasciatus* females ranged from 28 to 43.

The proportions killed are shown in Table 2. The control mortality of *An. arabiensis* females was 18%, while the treatment mortalities were 41% (boric acid), 45% (tolfenpyrad) and 48% (chlorfenapyr). The difference between the control and ATSB mortalities was significant for all three treatments (p<0.001). Treatment mortalities corrected for control using Abbott’s formula were 28% (boric acid), 33% (tolfenpyrad) and 37% (chlorfenapyr). As in the laboratory experiment, the proportions killed by each treatment were generally higher than the proportions observed with visible dye. The mortality of *An. arabiensis* males was slightly higher than for females but after correction for control the treatment mortalities were: 42% (boric acid), 29% (tolfenpyrad) and 29% (chlorfenapyr). The mortality of *Cx. quinquefasciatus* females was similar to that of *An. arabiensis* females across all three ATSB treatments (Table 2), and after correction for control the treatment mortalities for *Cx. quinquefasciatus* females were 39% (boric acid), 35% (tolfenpyrad) and 31% (chlorfenapyr). The difference between the control and ATSB mortalities were significant for all three treatments (p<0.05). There were no significant differences between the three ATSB treatments in the killing of *An. arabiensis* females or *Cx. quinquefasciatus* females (p>0.05).

**Table 2:** Results of experimental hut trial of three toxic sugar baits against *An. arabiensis* and *Cx. quinquefasciatus*. Numbers of female mosquitoes tested, the percentage dying, the percentage dyed with a red dye, and the percentage of dyed mosquitoes that died (and 95% confidence intervals) are presented.

			**Control**	**Chlorfenapyr (0.5%)**	**Boric acid (2%)**	**Tolfenpyrad (1%)**
***Anopheles arabiensis*** females						
	**Total caught**		97	100	104	109
	**Mortality (%)**		18 (11-26)^a^	48 (38-58)^b^	41 (32-51)^b^	45 (36-54)^b^
	**Mosquitoes with visible dye (%)**		25 (17-34)^a^	17 (11-26)^a^	15 (10-24)^a^	17 (11-26)^a^
	**Mortality of visibly dyed mosquitoes (%)**		13 (4-32)^a^	100^b^	94 (67-99)^c^	84 (61-95)^c^
	**Dead mosquitoes that were visibly dyed (%)**		18 (6-43)^a^	35 (23-50)^a^	35 (22-50)^a^	33 (21-47)^a^
***Anopheles arabiensis*** males						
	**Total caught**		172	165	210	213
	**Mortality (%)**		27 (21-34)^a^	58 (50-64)^b^	48 (41-54)^c^	48 (42-55)^c^
	**Mosquitoes with visible dye (%)**		19 (13-25)^a^	11 (7-17)^ab^	11 (8-16)^b^	10 (7-15)^b^
	**Mortality of visibly dyed mosquitoes (%)**		16 (7-33)^a^	89 (65-97)^b^	95 (73-99)^b^	95 (73-99)^b^
	**Dead mosquitoes that were visibly dyed (%)**		11 (5-25)^a^	17 (11-26)^a^	24 (17-33)^a^	19 (13-28)^a^
***Culex quinquefasciatus*** females						
	**Total caught**		43	42	35	28
	**Mortality (%)**		7 (2-20)^a^	43 (29-58)^b^	40 (25-57)^b^	36 (20-55)^b^
	**Mosquitoes with visible dye (%)**		12 (5-25)^a^	21 (12-36)^a^	29 (16-45)^a^	7 (2-25)^a^
	**Mortality of visibly dyed mosquitoes (%)**		0^a^	78 (42-94)^b^	80 (46-95)^b^	100^c^
	**Dead mosquitoes that were visibly dyed (%)**		0^a^	39 (20-62)^a^	57 (32-79)^a^	20 (5-54)^a^

If the superscript in a row is the same, there were no significant differences between the treatments (p>0.05)

The proportion of mosquitoes observed with visible dye ranged from 7% to 29% depending on the species or treatment (Table 2). The mortality rates of dyed mosquitoes in the three huts with insecticide were much higher, ranging from 78% to 100%, while the mortality of dyed mosquitoes in the control ranged between 12% and 16%. In huts with ATSB treatments, the proportion of dead mosquitoes with dye visible in their abdomens was at most 35% for *An. arabiensis* females, 24% for *An. arabiensis* males and 57% for *Cx. quinquefasciatus* females. 

## Discussion

Both the laboratory bioassay tests and the experimental hut trial demonstrate that insecticides originally developed to control sucking or chewing plant pests can also be used successfully as stomach poisons to kill medically important insects. Chlorfenapyr (pyrrole), tolfenpyrad (pyrazole), and boric acid (inorganic acid) are from diverse classes of oral insecticide yet all were successful in killing a high proportion of *An. arabiensis* and *Cx. quinquefasciatus* females in situations that mimicked domestic environments. ATSB constitutes a new mode of insecticide delivery that offers the prospect of harnessing, for the first time, new classes of active ingredient in the cause of malaria control and elimination. Conventional neurotoxic insecticides such as the pyrethroids, organophosphates and carbamates, while not tested here, may also have potential as ATSB. However it is probably judicious to restrict the range of ATSB active ingredients to the new classes of insecticide and to reserve the older conventional insecticides (that have residual contact efficacy) to LLINs and indoor residual spraying (IRS). 

It was observed in the laboratory bioassays that the proportions dying were considerably higher than the proportions that visibly contained dye, particularly for the chlorfenapyr and tolfenpyrad treatments. Either the mosquitoes were dying after tarsal contact with the ATSB surfaces or the dye is an imperfect indicator of mosquitoes that have ingested sufficient volumes of insecticide to induce mortality. Whilst chlorfenapyr is effective as a contact insecticide [17], tolfenpyrad is relatively inactive by tarsal contact, even at application rates as high as 1g/m^2^ and an exposure time of 10 minutes (Irish et al., unpublished). Boric acid acts mainly as a stomach poison [22] and has very low contact toxicity [23], so it seems unlikely that much of the insecticidal activity observed in the feeding bioassays was due to contact toxicity. Many mosquitoes that were observed feeding on the ATSB in the cage test and died shortly afterwards did not show visible dye in their abdomens. It seems that the dye assay lacks sensitivity and a high proportion of mosquitoes were dead after ingesting small volumes of ATSB undetectable by eye through the insect abdominal wall. A more sensitive assay might make use of radiolabel or other tracer chemicals.

Significantly higher proportions of mosquitoes died in the experimental huts containing ATSB treatments than in the control hut. Although the overall proportion of *An. arabiensis* females killed did not reach 50%, the level of mortality observed is still considerable and is not unusual for this species in huts. For example, hut trials of pyrethroid-treated bed nets against *An. arabiensis* rarely exceed 50% mortality in this area [21,24,25]. 

ATSB were not tested in the absence of mosquito nets. It is doubtful whether host-seeking mosquitoes would be diverted from blood feeding to ATSB stations were it not for the barrier of an ITN or LLIN [26]. It seems unlikely that indoor ATSB would provide protection in the absence of a net but this remains to be demonstrated. It is more likely that mosquitoes become diverted after expending time and energy trying to reach the host through the net. The combination of ATSB plus LLIN is a practical and conceptually sound way to control vector borne disease. As with other forms of indoor vector control, such as indoor residual spraying, there will be multiple opportunities to kill the mosquito after each gonotrophic cycle when it returns to the house to feed, which will have the cumulative effect of reducing the lifespan of mosquito population, an essential factor in malaria transmission control. The combination of ATSB and LLIN also has potential as a new tool for insecticide resistance management. One propounded method of resistance management is the simultaneous delivery of unrelated classes of insecticides either as mixtures on the same net or as LLIN and IRS in the same household, but both approaches have been a challenge owing to the limited number of insecticide classes which can be used for treated nets or IRS treatments [27,28]. The potential of ATSB to deliver classes of insecticide completely new to malaria control makes it ideal for controlling pyrethroid-resistant mosquitoes that survive initial contact with LLINs. An ATSB station should be a far cheaper and more targeted means of delivering insecticide than the application of insecticide to all wall surfaces as is required by IRS. 

The supply of safe and effective contact insecticides for use in public health is diminishing and the development of new residual insecticides just for malaria control is uneconomic for pesticide innovators [29-31]. The ATSB approach provides a route for delivering modern insecticides - developed through the more lucrative agricultural pipeline as crop protectants against chewing pests - to the public health sector as control agents of disease vectors. 

## Conclusion

ATSBs were effective in controlling *An. arabiensis* and *Cx. quinquefasciatus* in a simulated domestic setting. The optimization of bait stations for killing pyrethroid-resistant mosquitoes in the home environment has potential to lead to an effective and economic means of utilizing alternative insecticides that are otherwise unsuitable for malaria control. 
